# Efficacy of various plant-derived interventions in the prevention of radiation dermatitis in breast cancer patients: a systematic review and network meta-analysis of randomised controlled trials

**DOI:** 10.3389/fonc.2025.1657588

**Published:** 2025-10-22

**Authors:** Mingyu Li, Jianping Hao, Guoming Song, Ming Zhang, Bo Zhang, Yunran Hao, Lijun Zhao

**Affiliations:** Department of Radiation Therapy, The Second Affiliated Hospital of Xingtai Medical College, Xingtai, China

**Keywords:** breast cancer, radiation dermatitis, plant-derived substances, systematic review, network meta-analysis

## Abstract

**Objective:**

Radiation dermatitis (RD), a common adverse event among breast cancer patients undergoing post-surgical radiotherapy, may be mitigated through the application of plant-derived substances possessing radioprotective effects. However, comprehensive evaluations comparing the efficacy of different plant-derived compounds are not yet available. The objective of this study is to perform a network meta-analysis (NMA) to evaluate the efficacy of diverse plant-derived substances in preventing RD in patients with breast cancer.

**Methods:**

A systematic search was conducted to identify randomized controlled trials (RCTs) published up to April 2025 that investigated the use of plant-derived substances for the prevention of RD in patients with breast cancer. Two authors individually screened the articles, gathered pertinent information, and conducted quality assessments of the studies that were included.Data were synthesized and analyzed using Stata version 15.1.

**Results:**

In our NMA, we included 18 RCTs involving 2177 patients and 18 different treatment arms. Regarding the primary and secondary outcomes, Sylimarin derived from *Silybum marianum* L.(Milk thistle) (SUCRA = 0.934) and *Cichorium intybus* L.(Chicory) root extract (SUCRA = 0.72) demonstrated the greatest efficacy in mitigating the occurrence of grade ≥2 and grade ≥3 RD. Furthermore, Silymarin (RR = 0.05, 95% CI [0.00, 0.87]) exhibits greater efficacy compared with the standard of care (SOC) in preventing grade ≥2 RD. However, no intervention demonstrated superiority over SOC in preventing grade ≥3 RD.

**Conclusion:**

Silymarin has shown promise as treatment for the prevention of grade ≥2 RD in patients undergoing radiotherapy. Future studies with larger sample sizes are needed to substantiate the efficacy of various plant-derived substances.

**Systematic review registration:**

https://www.crd.york.ac.uk/prospero/, identifier CRD420251063723.

## 1 Introduction

Globally, breast cancer ranks as the most common malignant neoplasm in the female population (1), significantly impacting their health. Radiotherapy, an effective anti-tumor modality, plays a crucial role in reducing local recurrence following breast cancer surgery and extending patient survival.Radiation dermatitis (RD) is the primary adverse effect of this treatment, affecting 80–95% patients who receive radiotherapy for breast cancer (2). The direct or indirect cytotoxic effects of high-energy radiation cause oxidative stress, DNA damage, the release of inflammatory mediators, and mitochondrial impairment (3), ultimately leading to radiation-induced dermatitis. Clinical manifestations include alterations in skin pigmentation, alopecia, erythema, desquamation, ulceration, hemorrhage, and necrosis (4).To mitigate the occurrence of RD, extensive research has been conducted over the years. This body of research has focused on advancing radiotherapy technology (5, 6), investigating optimal dose-fractionation schedules (7, 8), exploring photobiomodulation therapy (9, 10) and assessing various topical medications. Although these interventions can reduce the occurrence of RD and mitigate symptoms including itching and pain, their overall efficacy remains limited, and the prolonged use of some medications may lead to adverse effects (11).

Polysaccharides, glycoproteins, amino acids, and pectins are active substances found in plant extracts such as *Aloe vera* (L.) Burm.f. (Aloe vera), *Azadirachta indica* (Azadirachta indica), and *Cucumis sativus* L. (Cucumis sativus). These substances can form a hydrating barrier on the skin, thereby providing moisturization and soothing effects that help alleviate skin damage associated with dry desquamation (12, 13).Catechins, flavonolignans, phenolic compounds, follicular acids can scavenge free radicals, especially reactive oxygen species (ROS) and reactive nitrogen species (RNS), increase the activities of superoxide dismutase (SOD), catalase (CAT) and glutathione peroxidase (GPX), and inhibit the production of inflammatory factors, such as tumor necrosis factor alpha (TNF-α), interleukin 6 (IL-6) and interleukin 1β (IL-1β), reducing inflammatory reactions (14–16). Ryan et al. (17), Zhao et al. (18), Karbasforooshan et al. (19), and Xie et al. (20) have demonstrated that active substances contained in plants, including *Silybum marianum* L.(Milk thistle), *Curcuma longa* L.(Turmeric), and *Camellia sinensis* (L.) Kuntze. (Tea plant) can effectively reduce the incidence of acute RD at grade 2 or higher, delay the onset of skin toxicity, and enhance wound healing. In terms of adverse reactions, allergies were more commonly associated with Aloe vera extract, while Turmeric (17) and Tea plant extract (18) scored slightly higher than controls on pain perception, but these differences did not achieve statistical significance. The overall tolerability of these interventions was good, and reports of adverse events leading to discontinuation were rare. Most current studies compare plant-derived interventions with the standard of care (SOC) or best supportive care. However, limited research exists comparing the efficacy of different plant-derived agents. As a result, the selection of interventions is frequently guided by anecdotal evidence from clinical practice rather than empirical clinical data.

This investigation aims to offer valuable insights for clinical decision-making by using a network meta-analysis (NMA) to evaluate the efficacy of various plant-derived substances in reducing RD in breast cancer patients using probability ranking.

## 2 Methods

### 2.1 Study protocol

This study followed the PRISMA-NMA: Preferred Reporting Items for Systematic Reviews and Meta-Analyses incorporating Network Meta-Analyses of health care interventions, and was registered in the PROSPERO (CRD420251063723).

### 2.2 Literature retrieval

PubMed, Cochrane Library, Web of Science, and Embase were independently searched by two authors (ML and JH), covering the period from database inception to April 2025. The search strategy included the terms “Breast cancer” AND (“Plant active substances” OR “Plant active ingredients” OR “Plant-Based Bioactive Compounds” OR “Plant extracts”OR”Phytochemicals” OR “Phytoestrogens” OR “Plant Growth Regulators” OR “xanthohumol D” OR “PC-Spes2”) AND “RCT”, applied as both free-text and MeSH terms.**Supplementary Table S1** delineates an example of the search outcomes obtained from PubMed. Furthermore, previously published systematic reviews and meta-analyses relevant to our research subject, as well as the reference lists of the studies included in our review, were examined to identify any potentially missed articles. When disagreements arose, the issues were addressed and resolved through consultation with a third author (MZ).

### 2.3 Inclusion and exclusion criteria

Eligibility criteria were based on the PICOS framework.

Inclusion criteria: (1) Population: Breast cancer patients receiving radiotherapy to the chest wall, breast, or regional lymph nodes after surgery, with no pre-existing skin lesions prior to radiotherapy. (2) Intervention: Administration of a single-plant extract, applied exclusively by the topical route, with the duration of the intervention clearly reported. (3) Comparator: SOC, including conventional medications (e.g., rhEGF, Trolamine Cream, Corticosteroids, Biafine or other moisturizing creams/gels), placebo, blank control, or conventional supportive care. (4) Outcomes: Primary outcome: Incidence of Grade ≥2 radiation dermatitis (RD), based on RTOG or CTCAE criteria (i.e., incidence of moist desquamation).Secondary outcome: Incidence of Grade ≥3 RD (RTOG/CTCAE). (5) Study design: RCT.

Exclusion criteria: (1) Non-randomized studies, including case reports, case series, cohort studies, case-control studies, or self-controlled trials. (2) Duplicate publications. (3) Studies with incomplete or missing data. (4) Full-text articles not available.

### 2.4 Data extraction

Authors ML and JH separately extracted information from the incorporated studies.The extracted information comprised the following: author, year of publication, country, sample size, tumor stage, radiotherapy dose, whether chemotherapy was administered prior to radiotherapy, evaluation criteria for RD, intervention measures, control measures, mode of application, and outcome indicators.

### 2.5 Quality assessment

The methodological quality of the included studies was evaluated using the Cochrane Risk of Bias Tool, and risk-of-bias(ROB) plots were generated using RevMan 5.2 software. The evaluation of bias was based on seven key criteria: generation of random sequences, allocation concealment, blinding of participants and researchers, blinding of outcome assessors, completeness of outcome data, selective reporting, and other potential sources of bias. Each criterion was assessed and categorized as having a low, unclear, or high risk of bias.Authors ML and GM conducted independent assessments of the risk of bias in the included studies. Any discrepancies were addressed through consultation with a third author (MZ).

### 2.6 Statistical analysis

We conducted a standard NMA using Stata 15.1 to ascertain the overall RR and corresponding 95% confidence interval (95% CI) for categorical data. The network evidence plot depicted direct comparisons among various interventions, with node size representing the sample size of each intervention and the thickness of connecting lines indicating the number of studies making direct comparisons. Funnel plots were employed to evaluate publication bias, and consistency tests were conducted when closed loops were present in the network evidence plot. Surfaces under the cumulative ranking curve (SUCRA) were computed to assess the relative efficacy of each intervention for both primary and secondary outcomes. SUCRA values provided a ranking based on the probability that each treatment has the highest efficacy. A league table was used to perform direct and indirect comparisons between treatments to evaluate their relative efficacy.

## 3 Results

### 3.1 Study selection

**Figure 1:**
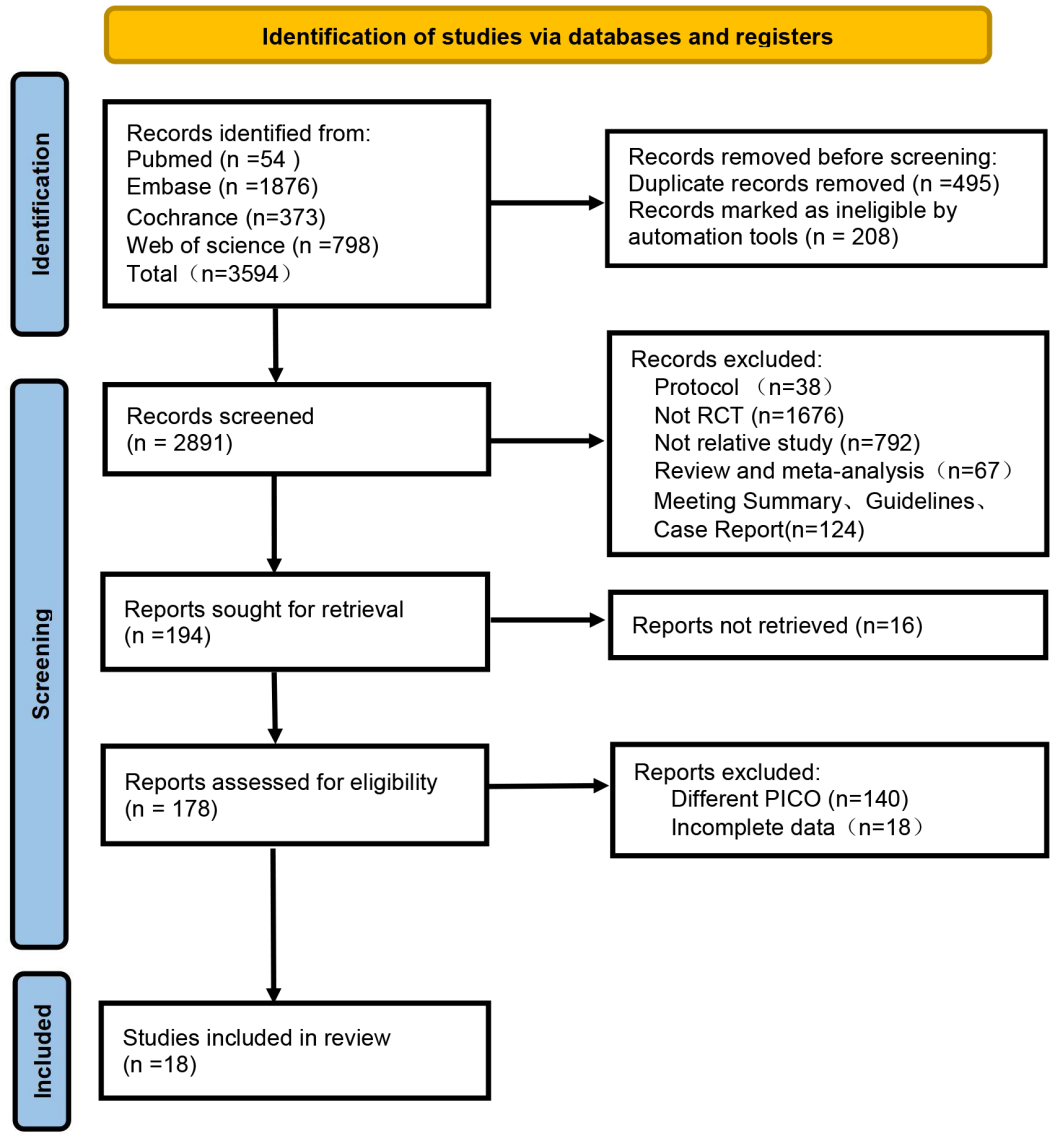
Flow diagram of the study selection process.

A total of 3, 594 articles were retrieved from the databases. Following the removal of duplicates and studies automatically marked as ineligible, 2, 891 articles remained. Based on title and abstract screening, 2, 697 articles were excluded, leaving 178 for full-text assessment. After evaluating the full texts, 18 studies satisfied the inclusion criteria and were incorporated into the systematic review. **Figure 1** illustrates the study selection process.

### 3.2 Characteristics of the included studies

The studies included in this analysis were disseminated between 2004 and 2025 and were performed across 10 Nations: France, Sweden, Canada, China, Italy, Iran, Thailand, Australia, the United States, and Brazil. Among the 2, 177 patients who underwent radiation therapy following breast cancer surgery, sample sizes ranged from 30 to 390 participants. A total of 17 interventions derived from plants were assessed in comparison with SOC, including *Calendula officinalis* L.(Calendula), *Boswellia serrata* Roxb. ex Colebr.(Boswellia)*, Nigella sativa* L.(Nigella)*, Centella asiatica* (L.) Urb. (Centella asiatica)*, Thunbergia laurifolia* Lindl.(Thunbergia)*, Chamomilla recutita.* (Chamomile)*, Plantago major* L.(Plantago major) leaf*, Avena sativa* L.(Avena)*, Olea europaea* L.(Olive) Oil*, and Glycyrrhiza glabra* L.(Licorice)*, Achillea millefolium* L.(Yarrow), Milk thistle, Turmeric, Tea plant, Cucumis sativus, Aloe vera, Chicory root.In studies where the interventions were Milk thistle, Turmeric, and Tea plant extracts, it was explicitly stated that the investigations used the principal monomeric constituents of these extracts—namely Silymarin, Curcumin and Epigallocatechin-3-gallate (EGCG), respectively.The timing of administration ranged from 2–3 days before to 1 month after the initiation of radiotherapy. A summary of the features for each study incorporated in this review is detailed in **Supplementary Table S2** (18, 19, 21–36).

### 3.3 Risk of bias in included studies and publication bias

The risk of bias assessment plots and summary plots for the included RCTs are shown in **Supplementary Figures S1, S2**. For blinding of outcome assessment and selective reporting, all trials included in the analysis provided detailed descriptions. Regarding random sequence generation, incomplete outcome data, and other potential sources of bias, one study each 16.7% (3 out of 18) did not report sufficient details and was therefore regarded as having an unclear risk. For blinding of participants and personnel, 33.3% (6 out of 18) were classified as unclear risk, and 5.6% (1 out of 18) as high risk, due to inadequate descriptions and lack of blinding procedures. Given the unclear reporting of allocation concealment, 33.3% (6 out of 18) of studies were classified as having an unclear risk of bias. Funnel plot analysis, as shown in **Supplementary Figure S3**, indicated no evidence of publication bias in this NMA, suggesting a balanced distribution of studies regardless of their outcomes.

### 3.4 Outcome indicators of network meta-analysis

**Figure 2:**
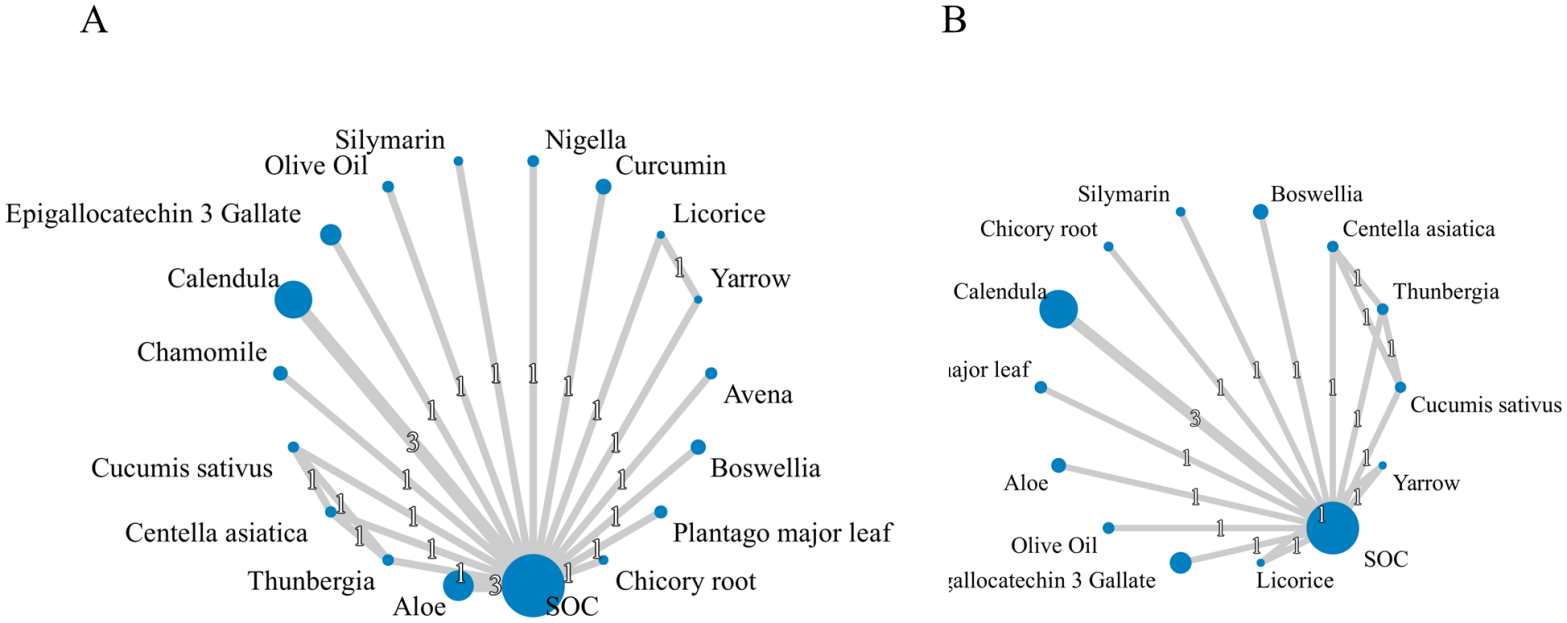
Network plots of available interventions for each outcome. **(A)** Incidence of grade ≥2 RD. **(B)** Incidence of grade ≥3 RD. The node size represents the sample size of each intervention and the thickness of connecting lines indicates the number of studies making direct comparisons.

**Figure 3:**
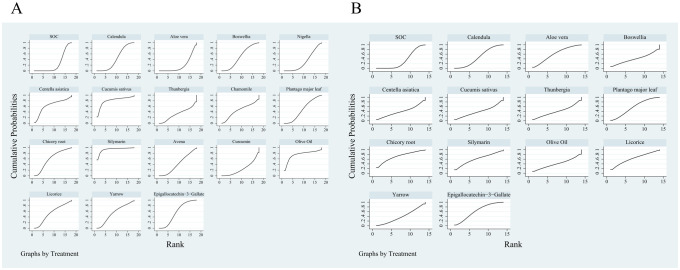
Cumulative ranking curves for two outcome indicators. **(A)** Incidence of grade ≥2 RD, **(B)** Incidence of grade ≥3 RD.

The results of the network plots are presented in **Figure 2**. The SUCRA (%) values for the two outcome indicators are illustrated in **Table 1**. Cumulative ranking curves for each outcome are summarized in **Figure 3**. Additionally, league tables are provided in **Supplementary Tables S3, S4**.

**Table 1:** Interventions’ SUCRA(%) regarding each outcomes.

Interventions	Incidence of grade≥2 RD	Incidence of grade≥3 RD
	SUCRA(%)	SUCRA(%)
SOC	25.0	32.8
*Calendula officinalis* L. (Calendula)	42.4	47.4
*Aloe vera* (L.) Burm.f. (Aloe vera)	19.4	60.6
*Boswellia serrata* Roxb. ex Colebr. (Boswellia)	49.1	40.8
*Nigella sativa* L. (Nigella)	32.7	-
*Centella asiatica* (L.) Urb. (Centella asiatica)	63.8	42.3
*Cucumis sativus* L. (Cucumis sativus)	80.5	39.6
*Thunbergia laurifolia* Lindl. (Thunbergia)	32.4	41.5
*Chamomilla recutita.* (Chamomile)	47.3	-
*Plantago major* L. (Plantago major) leaf	44.7	56.8
*Cichorium intybus* L. (Chicory) root	60.3	**72.0**
*Silybum marianum* L.(Milk thistle) (Sylimarin)	**93.4**	61.0
*Avena sativa* L.(Avena)	41.2	-
*Curcuma longa* L.(Turmeric) (Curcumin)	24.7	-
*Olea europaea* L.(Olive) Oil	74.3	41.6
*Glycyrrhiza glabra* L.(Licorice)	54.1	62.7
*Achillea millefolium* L.(Yarrow)	54.6	38.7
*Camellia sinensis* (L.) Kuntze.(Tea plant) (EGCG)	47.7	56.8

SUCRA values range from 0 to 100%. The higher the SUCRA value, and the closer to 100%, the higher the likelihood that intervention is in the top rank or one of the top ranks. The top interventions are in bold text.

#### 3.4.1 Primary outcome

The evidence network was constructed using data from 18 RCTs that analyzed the incidence of grade 2 or higher RD following radiotherapy (18, 19, 21–36). These trials included 18 different treatment arms and involved a total of 2, 177 breast cancer patients. Based on SUCRA values, Silymarin derived from Milk thistle was ranked as the most effective intervention (SUCRA = 0.934), while Aloe vera extract was ranked the least effective (SUCRA = 0.194).

The league table revealed that Silymarin was significantly more effective than the SOC, with a risk ratio (RR) of 0.05 (95% CI [0.00, 0.87]). It also demonstrated superiority over Aloe vera extract (RR = 0.05 [95% CI 0.00, 0.80]), Curcumin (RR = 0.05 [95% CI 0.00, 0.87]) and Nigella extract (RR = 0.06 [95% CI 0.00, 0.95]). No significant differences were found in direct or indirect comparisons the remaining interventions.

#### 3.4.2 Secondary outcome

A total of 12 RCTs (18, 19, 21, 22, 24, 26–29, 33, 34, 36), involving 14 treatment arms and 1, 473 breast cancer patients, reported the incidence of grade ≥3 RD. The SUCRA results indicated that Chicory root extract ranked highest in efficacy (SUCRA = 0.72), whereas SOC ranked lowest (SUCRA = 0.328).

As shown in the league table, none of the interventions demonstrated superiority over the SOC in preventing grade ≥3 RD. Additionally, statistical significance was not achieved in any direct or indirect comparisons among the various interventions.

#### 3.4.3 Heterogeneity and inconsistency

The treatment loops for the two outcomes were formed by a three-arm trial and a four-arm trial, respectively. Consequently, the NMA was considered to be consistent according to the definition.

## 4 Discussion

This NMA of 18 RCTs, integrating direct and indirect evidence, hierarchically ranks plant-derived interventions for RD prevention in breast-cancer radiotherapy. SUCRA values indicate that Silymarin preparations emerged as the optimal strategy for grade ≥2 RD, and demonstrated superiority over the SOC, whereas Chicory root extract achieved the highest probability for grade ≥3 RD prevention, although without statistically significant superiority over SOC. This analytic framework transcends traditional pairwise meta-analyses by quantifying each intervention’s likelihood of being the best, second-best, or subsequent ranks, thereby supplying clinicians with rank-ordered evidence that directly informs individualized therapeutic choices.

Oxidative stress is recognized as a crucial element in the development and advancement of radiation-induced skin reactions (RISRs) (37, 38). Natural antioxidant compounds extracted from herbs show significant potential in the management of RISRs (39–41). In our NMA, we assessed the efficacy of 17 distinct plant-derived interventions in mitigating RD, and found that Silymarin exhibited the greatest probability of being the optimal intervention for preventing grade ≥2 RD. The primary active component in Silymarin is a flavonoid mixture (42) which has been shown to significantly enhance the levels of cellular antioxidants such as superoxide dismutase (SOD) and catalase (CAT), inhibit lipid peroxidation, and effectively eliminate free radicals and reactive oxygen species (ROS) (43, 44), thereby attenuating oxidative stress and reducing RISRs.These mechanistic pathways have been validated *in vitro* (45). Our findings collectively substantiate the radioprotective potential of silymarin. A meta-analysis by Suyun et al. (46) published in 2023, supported the efficacy of Silymarin in preventing RD among breast cancer patients. However, this conclusion was based on only two studies involving a total of 141 patients, making it difficult to establish strong clinical validity. Further supporting evidence was provided by Becker-Schiebe et al. (47) and Karbasforooshan et al. (19) who reported that application of 0.25% Silymarin cream or its 1% gel significantly delayed the onset of skin toxicity. During the fifth week of treatment, the incidence of grade ≥2 RD in the Silymarin-treated groups was markedly lower than in the control groups (9.8% vs. 52% and 0% vs. 45%, respectively). These rates were also substantially lower than those reported in previously published studies (31%–50%) (48). Collectively, these findings underscore the promising role of Silymarin in preventing radiation dermatitis in breast cancer patients.

According to our ranking, Chicory root extract was evaluated as the most effective intervention for preventing RD of grade ≥3, even though it was not superior to the SOC. It contains bioactive constituents, including phenolic acids and sesquiterpene lactones, that exert anti-inflammatory and antioxidant effects (49, 50) via ROS scavenging, NF-κB signalling blockade, and Nrf2-mediated up-regulation of HO-1 and NQO1, together with modulation of pro-inflammatory cytokines (e.g., IL-6, TNF-α). Additionally, a naturally occurring hydrogel in Chicory forms a water-absorbing, transparent film that may promote wound healing (51). A study (29) assessing the impact of a 3% Chicory root extract on the incidence and severity of radiation dermatitis reported that participants in the Chicory root group did not develop grade 3 or grade 4 RD. Within our analysis, the efficacy of Chicory root extract was not statistically different from that of other interventions. However, considering that only one study on Chicory root extract was included in our NMA, further large-sample randomized controlled trials are needed to validate its efficacy.

External preparations of Silymarin and Chicory root extract remain limited to institutional compounding or research use. Their absence from the commercial market, poor accessibility, and inadequate safety data preclude routine inclusion in supportive care.In view of cost, availability, and safety, external preparations of Aloe vera, Calendula, and Chamomile are more suitable for primary medical institutions or patients with limited economic conditions, thereby providing references for the selection of interventions in different clinical scenarios.

Compared with grade ≥2 RD, the overall incidence of grade ≥3 RD is relatively lower. Particularly in the era of intensity-modulated radiotherapy (IMRT) and hypofractionated radiotherapy, the incidence rate with routine care ranges from 5% to 15% (27, 52). In our NMA, the overall incidence was 4.69% (69 out of 1, 473 patients). The occurrence of grade ≥3 RD is often associated with more complex, individualized risk factors (38) including BMI, smoking history, and diabetes.Treatments aimed at eliminating oxygen free radicals, supplying antioxidants, and providing moisturization are often insufficient to effectively prevent or reverse such severe skin injury. This may partly explain why none of the evaluated interventions demonstrated statistically significant efficacy in preventing grade ≥3 RD in our study.

### 4.1 Implications for clinical practice

This study indicates that Silymarin are more effective in mitigating RD, outperforming commonly used herbal treatments such as Aloe vera, Calendula, and Chamomile in clinical practice. This finding is consistent with previous studies (46), further emphasizing the role of plant-based antioxidants in reducing radiation-related skin injuries. With the optimization of purification techniques and continued exploration of optimal dosages and formulations, the therapeutic potential of these compounds can be maximized. This can not only improve treatment compliance and quality of life for cancer patients who are receiving radiotherapy, but may also be applicable in other contexts involving radiation-induced tissue damage.

### 4.2 Limitations of the study

Our NMA has several limitations. Although all included studies were RCTs, the number of eligible trials was limited and sample sizes were generally small, with Chicory root extract supported by only one trial, thereby compromising the robustness of the SUCRA rankings.Because of significant heterogeneity in the original data, we were unable to include additional outcome indicators in the quantitative analysis—such as pain, itching, erythema, and quality of life—which are clinically relevant to patients undergoing radiotherapy. Third, even though an intervention network was established, most comparisons within the NMA were indirect, with limited direct evidence and a restricted ability to assess inconsistency. Additionally, among the included RCTs, there was considerable variability in radiotherapy techniques(IMRT vs 3D-CRT), patient-related factors (BMI, smoking history, diabetes), and country-specific supportive care standards, constituting potential confounders that limit conclusive findings. Therefore, the NMA findings should be viewed with caution.

## 5 Conclusion

This NMA demonstrated that, among the 17 plant-derived interventions evaluated, Silymarin was the most effective in preventing grade ≥2 RD in breast cancer patients, as indicated by probability rankings. In terms of preventing grade ≥3 RD, the efficacy of Chicory root extract was not significantly different from that of the SOC, yet it ranked the highest. However, current evidence on the prevention and management of RD using plant-derived substances remains limited. Further multi-center, high-quality, large-sample RCTs are warranted to validate the therapeutic potential of these interventions in clinical practice.

## Data Availability

The original contributions presented in the study are included in the article/**Supplementary Material**. Further inquiries can be directed to the corresponding author.
