# Supplementary figures and images for: Efficacy of various plant-derived interventions in the prevention of radiation dermatitis in breast cancer patients: a systematic review and network meta-analysis of randomised controlled trials
Source: Front Oncol. 2025 Oct 22;15:1657588. doi: 10.3389/fonc.2025.1657588 (PMC12586008; doi:10.3389/fonc.2025.1657588)

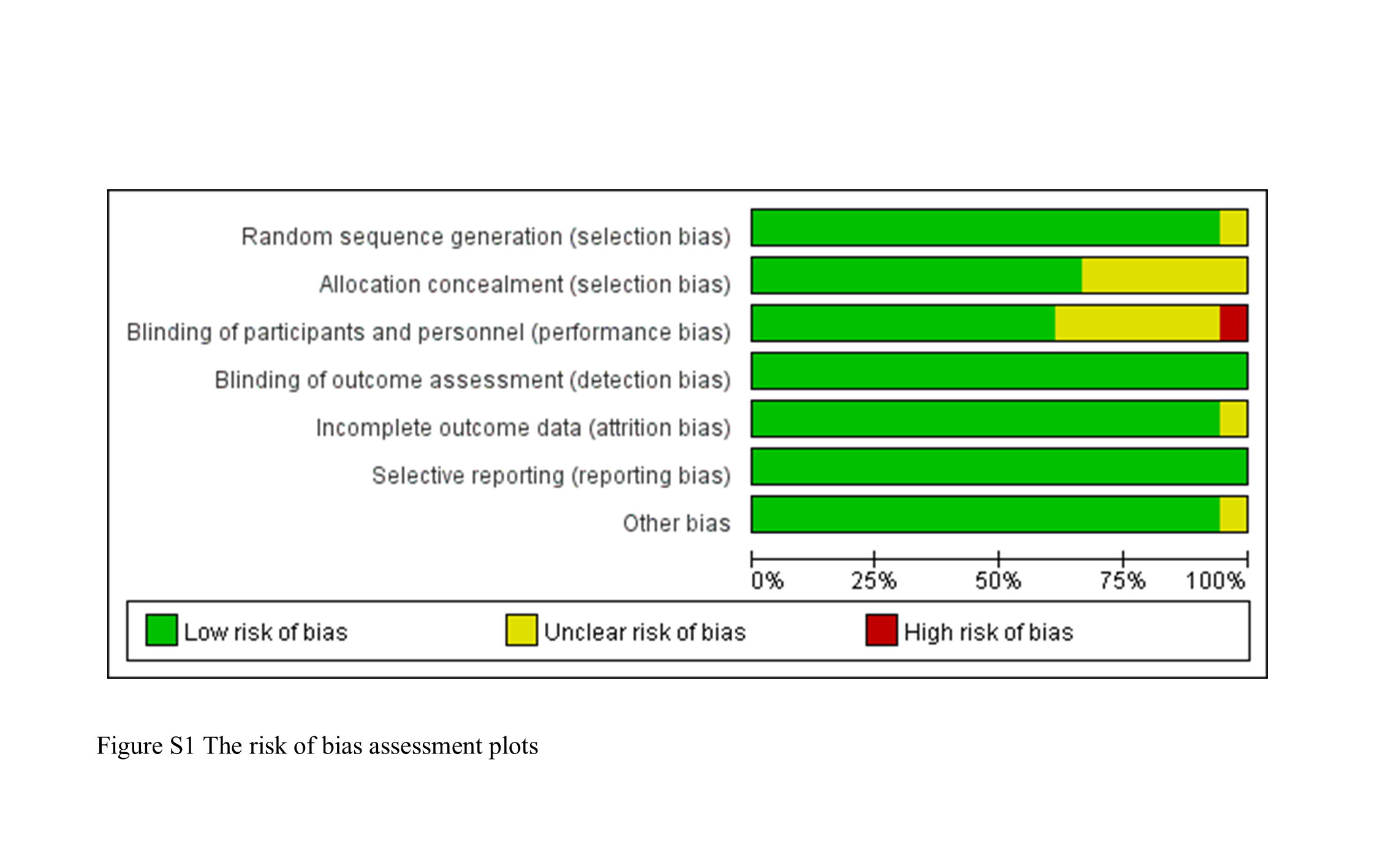

Supplement: Supplementary file 1 [file Image1.tif]

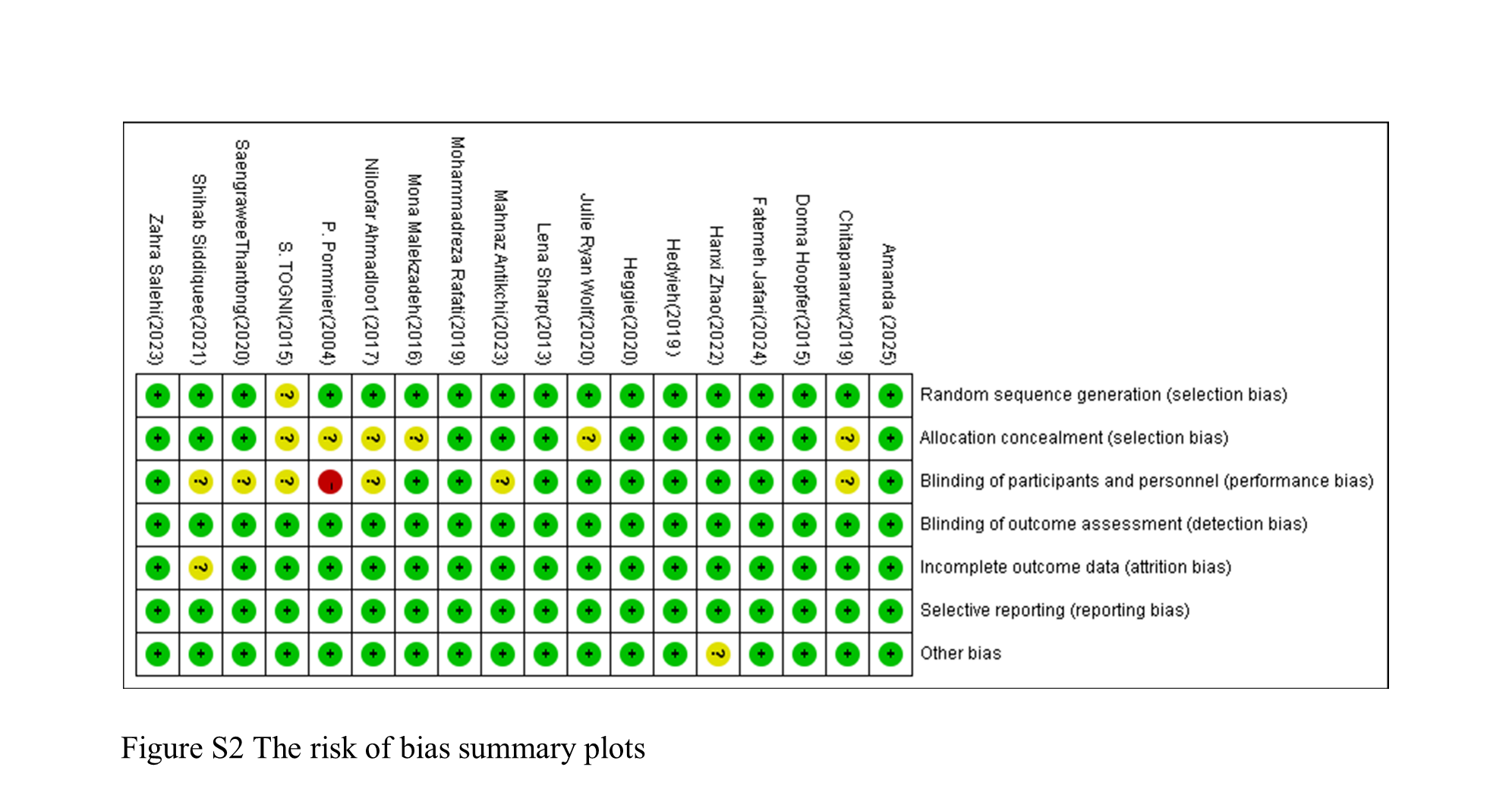

Supplement: Supplementary file 2 [file Image2.tif]

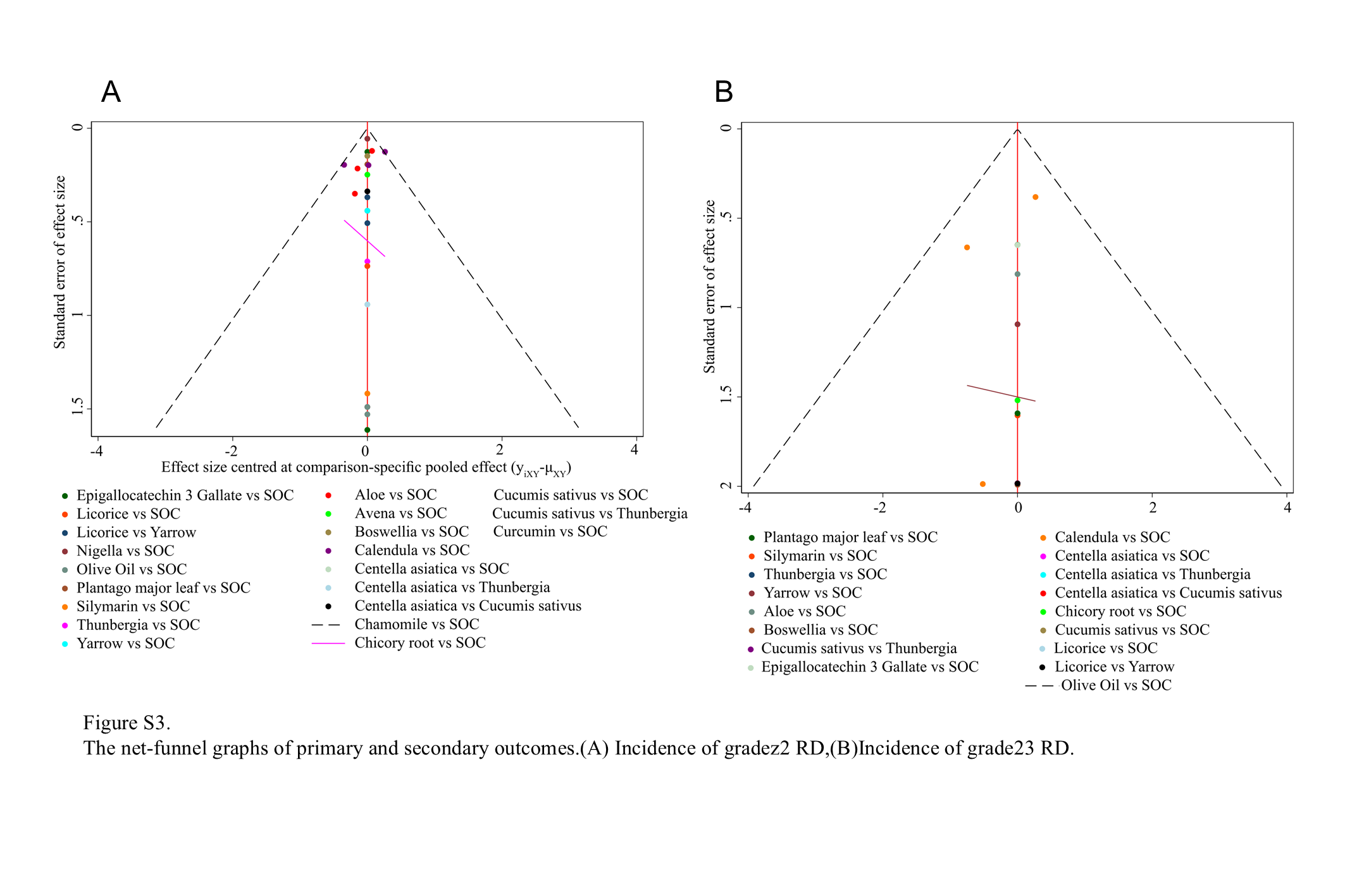

Supplement: Supplementary file 3 [file Image3.tif]
