# Supplementary material for: Efficacy of various plant-derived interventions in the prevention of radiation dermatitis in breast cancer patients: a systematic review and network meta-analysis of randomised controlled trials
Source: Front Oncol. 2025 Oct 22;15:1657588. doi: 10.3389/fonc.2025.1657588 (PMC12586008; doi:10.3389/fonc.2025.1657588)
Supplement: Supplementary file 4 [file Table1.doc]

Table S1 Systematic search outcomes retrieved from PubMed

| Search | PUBMED | Results |
| --- | --- | --- |
| #1 | ((((((((((((((((((((((((((((((((((((((Breast Neoplasms[MeSH Terms]) OR (Breast Neoplasms)) OR (Breast Neoplasm)) OR (Neoplasm, Breast)) OR (Neoplasms, Breast)) OR (Breast Tumors)) OR (Breast Tumor)) OR (Tumor, Breast)) OR (Tumors, Breast)) OR (Breast Cancer)) OR (Cancer, Breast)) OR (Cancer of Breast)) OR (Cancer of the Breast)) OR (Malignant Neoplasm of Breast)) OR (Breast Malignant Neoplasm)) OR (Breast Malignant Neoplasms)) OR (Malignant Tumor of Breast)) OR (Breast Malignant Tumor)) OR (Breast Malignant Tumors)) OR (Mammary Cancer)) OR (Cancer, Mammary)) OR (Cancers, Mammary)) OR (Mammary Cancers)) OR (Mammary Neoplasms, Human)) OR (Human Mammary Neoplasm)) OR (Human Mammary Neoplasms)) OR (Neoplasm, Human Mammary)) OR (Neoplasms, Human Mammary)) OR (Mammary Neoplasm, Human)) OR (Breast Carcinoma)) OR (Breast Carcinomas)) OR (Carcinoma, Breast)) OR (Carcinomas, Breast)) OR (Mammary Carcinoma, Human)) OR (Carcinoma, Human Mammary)) OR (Carcinomas, Human Mammary)) OR (Human Mammary Carcinomas)) OR (Mammary Carcinomas, Human)) OR (Human Mammary Carcinoma) | 566508 |
| #2 | (Plant active substances[MeSH Terms]) OR (Plant active substances) | 19680 |
| #3 | (Plant active ingredients[MeSH Terms]) OR (Plant active ingredients) | 12969 |
| #4 | (Plant-Based Bioactive Compounds[MeSH Terms]) OR (Plant-Based Bioactive Compounds) | 885 |
| #5 | (((((((((((((((((((((((((((Plant Extracts[MeSH Terms]) OR (Phytoestrogens[MeSH Terms])) OR (Drugs, Chinese Herbal[MeSH Terms])) OR (Plant Extracts)) OR (Phytoestrogens)) OR (Drugs, Chinese Herbal)) OR (Extracts, Plant)) OR (Plant Extract)) OR (Extract, Plant)) OR (Herbal Medicines)) OR (Medicines, Herbal)) OR (Plant Estrogens)) OR (Estrogens, Plant)) OR (Plant Estrogen)) OR (Estrogen, Plant)) OR (Phyto-Estrogen)) OR (Phyto Estrogen)) OR (Phytoestrogen)) OR (Estrogen-Like Plant Extracts)) OR (Estrogen Like Plant Extracts)) OR (Extracts, Estrogen-Like Plant)) OR (Plant Extracts, Estrogen-Like)) OR (Chinese Drugs, Plant)) OR (Chinese Herbal Drugs)) OR (Herbal Drugs, Chinese)) OR (Plant Extracts, Chinese)) OR (Chinese Plant Extracts)) OR (Extracts, Chinese Plant) | 359326 |
| #6 | ((((((((((((((((((((((((((((((((Phytochemicals[MeSH Terms]) OR (Biologically Active Compounds, Plant)) OR (Plant-Derived Compound)) OR (Compound, Plant-Derived)) OR (Plant Derived Compound)) OR (Dietary Phytochemical)) OR (Phytochemical, Dietary)) OR (Plant Bioactive Compound)) OR (Bioactive Compound, Plant)) OR (Compound, Plant Bioactive)) OR (Plant Biologically Active Compound)) OR (Dietary Phytochemicals)) OR (Phytochemicals, Dietary)) OR (Plant Bioactive Compounds)) OR (Bioactive Compounds, Plant)) OR (Compounds, Plant Bioactive)) OR (Plant Biologically Active Compounds)) OR (Plant-Derived Chemical)) OR (Chemical, Plant-Derived)) OR (Plant Derived Chemical)) OR (Bioactive Coumpounds, Plant)) OR (Coumpounds, Plant Bioactive)) OR (Plant Bioactive Coumpounds)) OR (Phytochemical)) OR (Phytonutrient)) OR (Plant-Derived Chemicals)) OR (Chemicals, Plant-Derived)) OR (Plant Derived Chemicals)) OR (Phytonutrients)) OR (Plant-Derived Compounds)) OR (Compounds, Plant-Derived)) OR (Plant Derived Compounds)) OR (Phytochemicals) | 93464 |
| #7 | ((((((((((((Phytoestrogens[MeSH Terms]) OR (Phytoestrogens)) OR (Plant Estrogens)) OR (Estrogens, Plant)) OR (Plant Estrogen)) OR (Estrogen, Plant)) OR (Phyto-Estrogen)) OR (Phyto Estrogen)) OR (Phytoestrogen)) OR (Estrogen-Like Plant Extracts)) OR (Estrogen Like Plant Extracts)) OR (Extracts, Estrogen-Like Plant)) OR (Plant Extracts, Estrogen-Like) | 17046 |
| #8 | (((((((((((((((((((((((((((Plant Growth Regulators[MeSH Terms]) OR (Florigen[MeSH Terms])) OR (Chlormequat[MeSH Terms])) OR (Cytokinins[MeSH Terms])) OR (Plant Growth Regulators)) OR (Florigen)) OR (Chlormequat)) OR (Cytokinins)) OR (Plant Hormone)) OR (Hormone, Plant)) OR (Phytohormones)) OR (Growth Regulators, Plant)) OR (Regulators, Plant Growth)) OR (Plant Hormones)) OR (Hormones, Plant)) OR (Plant Growth Regulator)) OR (Growth Regulator, Plant)) OR (Regulator, Plant Growth)) OR (Phytohormone)) OR (Flowering Hormone)) OR (Hormone, Flowering)) OR (Chlormequat Chloride)) OR (Chloride, Chlormequat)) OR (Chlorinecolinchloride)) OR (Chlorocholine Chloride)) OR (Chloride, Chlorocholine)) OR (Cycocel)) OR (Cytokinin) | 87274 |
| #9 | ((((xanthohumol D[MeSH Terms]) OR (PC-Spes2[MeSH Terms])) OR (xanthohumol D)) OR (PC-Spes2)) OR (Botanicals) | 36581 |
| #10 | #2 OR #3 OR #4 OR #5 OR #6 OR #7 OR #8OR #9 | 513692 |
| #11 | (((randomized controlled) OR (random)) OR (RCT)) OR (clinical trial) | 2215156 |
| #12 | #1 AND #10AND #11 | 547 |
