# Supplementary material for: Efficacy of various plant-derived interventions in the prevention of radiation dermatitis in breast cancer patients: a systematic review and network meta-analysis of randomised controlled trials
Source: Front Oncol. 2025 Oct 22;15:1657588. doi: 10.3389/fonc.2025.1657588 (PMC12586008; doi:10.3389/fonc.2025.1657588)
Supplement: Supplementary file 5 [file Table2.docx]

Table S2 Basic characteristics of the included studies

| Study | Sample size（n） | Average\Mean age | stage(%) | Chemotherapy before RT | RT Dose | RD criteria | Intervention | The form of intervention | Control |
| --- | --- | --- | --- | --- | --- | --- | --- | --- | --- |
| P. Pommier(2004)France(21) | 254 | 56.5\|55.1 | NR | YES:52.0%  NO:48.0% | 46-52Gy | RTOG | Calendula BID or more | ointment | SOC-Trolamine |
| Lena Sharp(2013)Sweden(22) | 390 | 58\|58 | NR | YES:37.5%  NO:62.5% | 42.56-50Gy | RTOG/EORTC | Calendula BID | cream | SOC-Essex cream |
| Donna Hoopfer(2015)Canada(23) | 153 | NR | NR | YES:57.6%  NO:42.4% | 45-50Gy | CSSP score | Aloe vera TID | gel | SOC-placebo |
| S. TOGNI(2015)Italy(24) | 114 | 58.5 | NR | NR | 50 Gy | RTOG | Boswellia BID | cream | SOC-placebo |
| Mohammadreza Rafati(2019)Iran(25) | 62 | 51.4\|50.2 | I-II:77.4% III:22.6% | YES:87.1%  NO:12.9% | 42.56–50 Gy | RTOG/EORTC | Nigella BID | gel | SOC-placebo |
| Saengrawee Thanthong(2020)Thailand(26) | 39 | 56.7\|56.8 | 0-II:47.5% III-IV:52.5% | YES:71.8%  NO:28.2% | 40-50Gy | RTOG/EORTC | Centella asiatica QD | cream | SOC-placebo |
|  | 38 | 55.1\|56.8 | 0-II:67.5% III-IV:32.5% | YES:81.8%  NO:18.2% | 40-50Gy | RTOG/EORTC | Cucumis sativus QD | cream | SOC-placebo |
|  | 40 | 56.5\|56.8 | 0-II:70.0% III-IV:30.0% | YES:79.2%  NO:20.8% | 40-50Gy | RTOG/EORTC | Thunbergia QD | cream | SOC-placebo |
| Shihab Siddiquee(2021)Australia(27) | 76 | 56\|60 | NR | YES:41%  NO or NR:59% | 40-60Gy | RTOG | Calendula QD | lotion | SOC-Placebo |
| Zahra Salehi(2023)Iran(28) | 73 | 48.16\|51.15 | T1-T2:86.3% T3-T4:13.7%  N-:31.5% N+:68.5% | NR | 50 Gy | RTOG/ CTCAE | Plantago major leaf BID | cream | SOC- placebo |
| Fatemeh Jafari(2024)Iran(29) | 50 | 52.6 | NR | NR | NR | RTOG | Chicory Root BID | gel | SOC-placebo |
| Amanda Gomes de Meneses(2025)Brazil(30) | 99 | 56\|51 | NR | NR | 44.1\|43.7Gy | GRAL | Chamomile BID | liposomal gel | SOC-placebo |
| Hedyieh Karbasforooshan(2019）Iran(19) | 40 | 49.5\|47.3 | II:42.5% III-IV:47.5% | NR | 50 Gy | RTOG/ CTCAE | Silymarin QD | gel | SOC-placebo |
| Mahnaz Antikchi(2023)Iran(31) | 66 | NR | NR | YES:98.5%  NO:1.5% | 50-60Gy | RTOG | Avena TID | cream | SOC-placebo |
| Julie Ryan Wolf(2020)Thailand(32) | 111 | 59.8\|59.8 | DCIS-II:86.4% III-IV:13.6% | YES:48.8%  NO:51.2% | 44-66 Gy | RDS/CTCAE | Curcumin TID | gel | SOC-placebo |
| Niloofar Ahmadloo1(2017)USA(33) | 100 | 48\|49.1 | NR | NR | 50Gy | ARMS | Aloe vera BID | gel | SOC-no treament |
| Chitapanarux(2019)Australia(34) | 62 | 55\|56 | I-II:51.5% III-IV:48.5% | YES:95.5%  NO:4.5% | 42.4Gy | RTOG | Olive Oil BID | oil | SOC-no treament |
| Heggie（2020）Australia(35) | 208 | 56\|60 | T1-T2:96.6% T3:1.4%  Unknow: 2% | YES:95.5%  NO:4.5% | 50.0-64.1Gy | Morbidity Rating Scale | Aloe vera TID | gel | SOC-aqueous cream |
| Mona Malekzadeh（2016）Iran(36) | 21 | 47.53\|46.79 | NR | NR | 50 Gy | RTOG | Licorice NR | cream | SOC-placebo |
|  | 21 | 45\|46.79 | NR | NR | 50 Gy | RTOG | Yarrow NR | cream | SOC-placebo |
| Hanxi Zhao（2022）China(18) | 165 | 46.5\|48.4 | T1-T2:87.3% T3-T4:12.7%  N-:13.3% N+:86.7% | NR | 50 Gy-60Gy | RTOG | Epigallocatechin-3-Gallate  NR | solution | SOC-placebo |

Abbreviations:RTOG,Radiation Therapy Oncology Group;EORTC,European Organization for Research and Treatment of Cancer;CSSP,Catterall skin scoring profile; CTCAE,National Cancer Institute Common Terminology for Adverse Events; RD,Radiodermatitis;RDS:Radiation dermatitis severity;NR:not reported;RT,Radiation Therapy;GRAL,Graduação da Radio dermatite Aguda;ARMSC:Acute Radiation Morbidity Score Criteria;MRS;Morbidity Rating Scale;SOC:standard of care.
