# Supplementary material for: Efficacy of various plant-derived interventions in the prevention of radiation dermatitis in breast cancer patients: a systematic review and network meta-analysis of randomised controlled trials
Source: Front Oncol. 2025 Oct 22;15:1657588. doi: 10.3389/fonc.2025.1657588 (PMC12586008; doi:10.3389/fonc.2025.1657588)
Supplement: Supplementary file 6 [file Table3.docx]

Table S3 League table with network meta-analysis (NMA) estimates for primary outcome

| Silymarin |  |  |  |  |  |  |  |  |  |  |  |  |  |  |  |  |  |
| --- | --- | --- | --- | --- | --- | --- | --- | --- | --- | --- | --- | --- | --- | --- | --- | --- | --- |
| 0.34 (0.01,20.08) | Cucumis sativus*.* |  |  |  |  |  |  |  |  |  |  |  |  |  |  |  |  |
| 0.26 (0.00,16.21) | 0.76 (0.01,51.78) | Olive Oil |  |  |  |  |  |  |  |  |  |  |  |  |  |  |  |
| 0.12 (0.00,3.48) | 0.34 (0.01,8.30) | 0.45 (0.01,15.86) | Centella asiatica |  |  |  |  |  |  |  |  |  |  |  |  |  |  |
| 0.09 (0.00,1.61) | 0.25 (0.01,5.34) | 0.33 (0.01,7.53) | 0.73 (0.09,5.68) | Chicory root |  |  |  |  |  |  |  |  |  |  |  |  |  |
| 0.08 (0.00,1.29) | 0.22 (0.01,4.29) | 0.29 (0.01,6.06) | 0.63 (0.09,4.39) | 0.87 (0.34,2.20) | Epigallocatechin-3-Gallate |  |  |  |  |  |  |  |  |  |  |  |  |
| 0.08 (0.00,1.53) | 0.23 (0.01,5.06) | 0.30 (0.01,7.13) | 0.67 (0.08,5.48) | 0.91 (0.26,3.17) | 1.05 (0.37,2.99) | Licorice |  |  |  |  |  |  |  |  |  |  |  |
| 0.08 (0.00,1.53) | 0.23 (0.01,5.06) | 0.30 (0.01,7.13) | 0.67 (0.08,5.48) | 0.91 (0.26,3.17) | 1.05 (0.37,2.99) | 1.00 (0.35,2.89) | Yarrow |  |  |  |  |  |  |  |  |  |  |
| 0.07 (0.00,1.18) | 0.20 (0.01,3.93) | 0.26 (0.01,5.55) | 0.58 (0.08,4.03) | 0.79 (0.31,2.03) | 0.91 (0.48,1.75) | 0.87 (0.30,2.48) | 0.87 (0.30,2.48) | Boswellia |  |  |  |  |  |  |  |  |  |
| 0.07 (0.00,1.19) | 0.20 (0.01,3.95) | 0.26 (0.01,5.58) | 0.58 (0.08,4.07) | 0.79 (0.30,2.08) | 0.91 (0.45,1.82) | 0.86 (0.29,2.54) | 0.86 (0.29,2.54) | 1.00 (0.49,2.03) | Plantago major leaf |  |  |  |  |  |  |  |  |
| 0.07 (0.00,1.71) | 0.21 (0.01,5.63) | 0.27 (0.01,7.90) | 0.60 (0.05,6.66) | 0.82 (0.15,4.51) | 0.95 (0.20,4.51) | 0.90 (0.15,5.26) | 0.90 (0.15,5.26) | 1.04 (0.22,4.98) | 1.05 (0.21,5.10) | Chamomile |  |  |  |  |  |  |  |
| 0.06 (0.00,1.06) | 0.18 (0.01,3.52) | 0.24 (0.01,4.97) | 0.53 (0.08,3.56) | 0.73 (0.31,1.72) | 0.84 (0.49,1.43) | 0.79 (0.30,2.13) | 0.79 (0.30,2.13) | 0.92 (0.53,1.60) | 0.92 (0.50,1.69) | 0.88 (0.19,4.02) | Calendula |  |  |  |  |  |  |
| 0.06 (0.00,1.11) | 0.18 (0.01,3.70) | 0.24 (0.01,5.23) | 0.53 (0.07,3.85) | 0.73 (0.26,2.01) | 0.84 (0.39,1.79) | 0.80 (0.26,2.45) | 0.80 (0.26,2.45) | 0.92 (0.42,1.99) | 0.92 (0.41,2.08) | 0.88 (0.18,4.42) | 1.00 (0.51,1.98) | Avena |  |  |  |  |  |
| 0.05 (0.00,1.23) | 0.15 (0.01,2.90) | 0.20 (0.01,5.68) | 0.44 (0.07,2.91) | 0.61 (0.12,3.18) | 0.70 (0.15,3.17) | 0.66 (0.12,3.72) | 0.66 (0.12,3.72) | 0.77 (0.17,3.50) | 0.77 (0.17,3.58) | 0.74 (0.09,5.86) | 0.84 (0.19,3.65) | 0.83 (0.17,4.00) | Thunbergia |  |  |  |  |
| **0.06 (0.00,0.95)** | 0.16 (0.01,3.17) | 0.21 (0.01,4.48) | 0.47 (0.07,3.23) | 0.65 (0.26,1.59) | 0.75 (0.41,1.35) | 0.71 (0.26,1.96) | 0.71 (0.26,1.96) | 0.82 (0.44,1.51) | 0.82 (0.43,1.59) | 0.78 (0.17,3.67) | 0.89 (0.55,1.45) | 0.89 (0.43,1.84) | 1.07 (0.24,4.76) | Nigella |  |  |  |
| **0.05 (0.00,0.87)** | 0.15 (0.01,2.90) | 0.20 (0.01,4.10) | 0.44 (0.07,2.91) | 0.61 (0.27,1.37) | 0.70 (0.45,1.09) | 0.66 (0.26,1.70) | 0.66 (0.26,1.70) | 0.77 (0.48,1.23) | 0.77 (0.45,1.31) | 0.74 (0.17,3.27) | 0.84 (0.62,1.12) | 0.83 (0.45,1.54) | 1.00 (0.24,4.24) | 0.94 (0.64,1.38) | SOC |  |  |
| **0.05 (0.00,0.87)** | 0.14 (0.01,2.90) | 0.18 (0.01,4.08) | 0.40 (0.05,3.06) | 0.55 (0.18,1.67) | 0.63 (0.26,1.53) | 0.60 (0.18,2.02) | 0.60 (0.18,2.02) | 0.70 (0.28,1.70) | 0.70 (0.28,1.76) | 0.67 (0.13,3.56) | 0.76 (0.34,1.71) | 0.76 (0.29,2.01) | 0.91 (0.18,4.64) | 0.85 (0.36,1.99) | 0.91 (0.42,1.94) | Curcumin |  |
| **0.05 (0.00,0.80)** | 0.14 (0.01,2.68) | 0.18 (0.01,3.78) | 0.40 (0.06,2.71) | 0.55 (0.23,1.32) | 0.63 (0.37,1.10) | 0.60 (0.22,1.63) | 0.60 (0.22,1.63) | 0.70 (0.39,1.23) | 0.70 (0.38,1.30) | 0.67 (0.15,3.07) | 0.76 (0.49,1.16) | 0.76 (0.38,1.51) | 0.91 (0.21,3.98) | 0.85 (0.51,1.41) | 0.91 (0.66,1.25) | 1.00 (0.44,2.28) | Aloe vera |

Comparisons of incidence of ≥Grade 2 RD of different interventions were shown and should be read from left to right. The effectiveness estimate is located at the intersection of the columndefining treatment and the row defining treatment. Incidence of ≥Grade 2 RD estimates are presented in odds ratio (RR) with the 95% Credible Intervals (CrI), a RR below 1.0 favors the rowdefining intervention (less presence of incidence of ≥Grade 2 RD, means the top-left treatment is better).Significant findings are highlighted in boldface.
