# Supplementary material for: Efficacy of various plant-derived interventions in the prevention of radiation dermatitis in breast cancer patients: a systematic review and network meta-analysis of randomised controlled trials
Source: Front Oncol. 2025 Oct 22;15:1657588. doi: 10.3389/fonc.2025.1657588 (PMC12586008; doi:10.3389/fonc.2025.1657588)
Supplement: Supplementary file 7 [file Table4.docx]

Table S4 League table with network meta-analysis (NMA) estimates for secondary outcome

| Chicory root |  |  |  |  |  |  |  |  |  |  |  |  |  |
| --- | --- | --- | --- | --- | --- | --- | --- | --- | --- | --- | --- | --- | --- |
| 0.61 (0.01,52.85) | Licorice |  |  |  |  |  |  |  |  |  |  |  |  |
| 0.49 (0.02,15.15) | 0.80 (0.02,27.88) | Epigallocatechin-3-Gallate |  |  |  |  |  |  |  |  |  |  |  |
| 0.57 (0.01,50.25) | 0.93 (0.01,89.97) | 1.17 (0.03,41.68) | Silymarin |  |  |  |  |  |  |  |  |  |  |
| 0.48 (0.01,16.79) | 0.78 (0.02,30.78) | 0.97 (0.09,10.02) | 0.83 (0.02,33.72) | Aloe vera |  |  |  |  |  |  |  |  |  |
| 0.41 (0.01,12.66) | 0.67 (0.02,23.31) | 0.84 (0.10,7.01) | 0.72 (0.02,25.55) | 0.86 (0.08,8.84) | Plantago major leaf |  |  |  |  |  |  |  |  |
| 0.29 (0.01,7.33) | 0.47 (0.02,13.59) | 0.59 (0.10,3.53) | 0.51 (0.02,14.92) | 0.61 (0.08,4.61) | 0.71 (0.12,4.22) | Calendula |  |  |  |  |  |  |  |
| 0.19 (0.00,29.10) | 0.31 (0.00,51.58) | 0.39 (0.01,27.17) | 0.33 (0.00,56.16) | 0.40 (0.01,31.06) | 0.47 (0.01,32.47) | 0.66 (0.01,39.27) | Olive Oil |  |  |  |  |  |  |
| 0.18 (0.00,27.45) | 0.29 (0.00,48.65) | 0.36 (0.01,25.69) | 0.31 (0.00,52.96) | 0.37 (0.00,29.35) | 0.43 (0.01,30.69) | 0.62 (0.01,37.13) | 0.93 (0.00,258.74) | Boswellia |  |  |  |  |  |
| 0.19 (0.00,29.08) | 0.31 (0.00,51.54) | 0.39 (0.01,27.15) | 0.33 (0.00,56.11) | 0.40 (0.01,31.03) | 0.47 (0.01,32.44) | 0.66 (0.01,39.23) | 1.00 (0.00,274.40) | 1.07 (0.00,296.82) | Thunbergia |  |  |  |  |
| 0.18 (0.00,28.13) | 0.30 (0.00,49.85) | 0.38 (0.01,26.26) | 0.32 (0.00,54.28) | 0.39 (0.00,30.01) | 0.45 (0.01,31.38) | 0.64 (0.01,37.95) | 0.97 (0.00,265.45) | 1.04 (0.00,287.14) | 0.97 (0.02,51.23) | Centella asiatica |  |  |  |
| 0.18 (0.00,27.18) | 0.29 (0.00,48.17) | 0.36 (0.01,25.37) | 0.31 (0.00,52.45) | 0.37 (0.00,29.00) | 0.44 (0.01,30.32) | 0.62 (0.01,36.66) | 0.94 (0.00,256.50) | 1.00 (0.00,277.46) | 0.94 (0.02,49.49) | 0.97 (0.02,51.12) | Cucumis sativus |  |  |
| 0.20 (0.00,9.50) | 0.33 (0.01,8.35) | 0.42 (0.03,6.44) | 0.36 (0.01,18.90) | 0.43 (0.02,7.78) | 0.50 (0.03,7.70) | 0.71 (0.06,8.47) | 1.07 (0.01,104.71) | 1.15 (0.01,113.51) | 1.07 (0.01,104.62) | 1.11 (0.01,108.06) | 1.15 (0.01,111.74) | Yarrow |  |
| 0.19 (0.01,4.16) | 0.31 (0.01,7.77) | 0.39 (0.09,1.75) | 0.33 (0.01,8.54) | 0.40 (0.07,2.38) | 0.47 (0.10,2.09) | 0.66 (0.25,1.74) | 1.00 (0.02,53.02) | 1.07 (0.02,57.58) | 1.00 (0.02,52.96) | 1.03 (0.02,54.70) | 1.07 (0.02,56.56) | 0.93 (0.09,9.19) | SOC |

Comparisons of incidence of ≥Grade3 RD of different interventions were shown and should be read from left to right. The effectiveness estimate is located at the intersection of the columndefining treatment and the row defining treatment. Incidence of ≥Grade3 RD estimates are presented in risk ratio (RR) with the 95% Credible Intervals (CrI), a RR below 1.0 favors the rowdefining intervention (less presence of incidence of ≥Grade3 RD, means the top-left treatment is better).Significant findings are highlighted in boldface.
